# High-Resolution and Robust One-Bit Direct-of-Arrival Estimation via Reweighted Atomic Norm Estimation

**DOI:** 10.3390/s24185936

**Published:** 2024-09-13

**Authors:** Rui Li, Jianchao Yang, Zheng Dai, Xingyu Lu, Ke Tan, Weimin Su

**Affiliations:** School of Electronic and Optical Engineering, Nanjing University of Science and Technology (NJUST), Nanjing 210094, China; li_rui@njust.edu.cn (R.L.); daizheng@njust.edu.cn (Z.D.); ee_luxingyu@njust.edu.cn (X.L.); tank@njust.edu.cn (K.T.); suweimin@mail.njust.edu.cn (W.S.)

**Keywords:** one-bit quantization, direction-of-arrival estimation, atomic norm minimization

## Abstract

In recent years, one-bit quantization has attracted widespread attention in the field of direction-of-arrival (DOA) estimation as a low-cost and low-power solution. Many researchers have proposed various estimation algorithms for one-bit DOA estimation, among which atomic norm minimization algorithms exhibit particularly attractive performance as gridless estimation algorithms. However, current one-bit DOA algorithms with atomic norm minimization typically rely on approximating the trace function, which is not the optimal approximation and introduces errors, along with resolution limitations. To date, there have been few studies on how to enhance resolution under the framework of one-bit DOA estimation. This paper aims to improve the resolution constraints of one-bit DOA estimation. The log-det heuristic is applied to approximate and solve the atomic norm minimization problem. In particular, a reweighted binary atomic norm minimization with noise assumption constraints is proposed to achieve high-resolution and robust one-bit DOA estimation. Finally, the alternating direction method of multipliers algorithm is employed to solve the established optimization problem. Simulations are conducted to demonstrate that the proposed algorithm can effectively enhance the resolution.

## 1. Introduction

Direction-of-arrival (DOA) estimation plays an important role in various fields, such as wireless communications, radar, and sonar systems, as it enables the localization and tracking of targets [1,2]. However, most DOA estimation methods typically rely on high-resolution analog-to-digital converters (ADCs), which are costly and power-intensive [3]. In many modern applications, such as large-scale antenna arrays [4,5], the deployment of high-resolution ADCs is prohibitively expensive. As a result, one-bit quantization has gained considerable attention as a low-cost, low-power solution for DOA estimation [3].

There has been extensive research on the use of one-bit ADCs for DOA estimation. Sparse arrays with one-bit quantizers are found to be as good as uniform linear arrays (ULAs) with unquantized data, and it has been proven that with one-bit quantization, nested and coprime arrays can still resolve more signal sources than the number of sensors, provided that the signals are uncorrelated [6]. The reduction in spectral efficiency due to low-precision ADCs is acceptable, when operating at a low-to-moderate signal-to-noise ratio (SNR) with plentiful bandwidth [7], and the Cramér–Rao bound (CRB) of the one-bit signal is π/2 times the CRB of the unquantized signal [8,9]. However, one-bit quantization still poses challenges. Due to the fact that one-bit signals only preserve the sign information and the relationship between the covariance of the unquantized signal and the covariance of the one-bit quantized signals is non-linear [10], many DOA estimation algorithms based on covariance analysis suffer from significant performance degradation.

To avoid the aforementioned problems, sparse recovery algorithms are introduced in one-bit DOA estimation [11,12,13,14,15,16,17]. Sparse recovery algorithms use the inherent sparsity of signals in the spatial domain, and do not require knowledge of the covariance matrices [18]. The sparse recovery algorithms also offer the advantages of low-snapshot estimation and robustness to correlated signals [19]. The binary iterative hard thresholding algorithm is extended to the complex-valued multi-snapshot case, which is well suited for DOA estimation scenarios with multiple antenna elements and limited snapshots [11]. An improved fixed-point continuation $\ell_{1}$ reconstruction algorithm is developed to enable its application of one-bit complex-valued signal DOA estimation [12]. The one-bit DOA estimation problem is also transformed into a maximum likelihood-based optimization problem with a row-sparse matrix, incorporating an $\ell_{2,1}$ norm regularization term to enhance the estimation accuracy [17]. Besides the above grid-based sparse recovery algorithms, gridless DOA estimation methods are also proposed through the exploration of atomic norm minimization (ANM) in [20,21,22,23,24] to avoid the grid mismatch issue.

ANM methods treat DOA estimation as a sparse reconstruction problem using a continuous, infinite dictionary, and recover the DOAs by solving a semidefinite programming (SDP) problem. Reference [13] first applies binary ANM (BANM) to the one-bit measurements, and proposes a dual polynomial approach to achieve continuous frequency estimation. The frequencies can be estimated in the continuous frequency domain, overcoming the grid mismatch issue in frequency estimation. A BANM DOA estimation method based on sparse linear arrays is also proposed, and the alternating direction method of multipliers (ADMM) is utilized to accelerate the implementation [14]. An algorithm based on accelerated proximal gradients to solve the BANM optimization problem is developed in [15]. However, these methods use the trace function to approximate the rank function, which is a loose approximation and exhibits a discrepancy, similar to the difference between the $\ell_{1}$-norm and the $\ell_{0}$-norm [22], which limits the achievable resolution due to the poorer fitting performance [25].

To improve the resolution of existing gridless one-bit DOA estimation, the binary atomic $\ell_{0}$-norm minimization problem is built and approximated in this paper by the log-det heuristic instead of the trace function approximation. In particular, our contributions are highlighted as follows:

- To achieve higher resolution than the atomic $\ell_{1}$ norm, we develop a new optimization model that combines the atomic $\ell_{0}$ norm with a regularization term for the sign consistency of one-bit received signals, representing a generalization of atomic $\ell_{0}$ norm minimization in a one-bit environment.
- We incorporate a noise constraint into the proposed optimization model, significantly reducing the impact of noise on the atomic $\ell_{0}$ norm and thereby enhancing the robustness of the optimization model.
- Rank approximation and the majority minimization (MM) principle are utilized to transform the formulated NP-hard optimization model into a convex optimization problem. Additionally, we derive the solution steps using the alternating direction method of multipliers (ADMM) algorithm.

The content of the paper is arranged as follows. Section 2 introduces the model of one-bit signal, and the principle of atomic norm minimization algorithm is also presented. In Section 3, the robust reweighted binary atomic norm minimization optimization method is proposed. Section 4 derives the solution of the ADMM algorithm for the proposed optimization problem, and provides the detailed algorithmic steps. Section 5 presents the numerical simulations and Section 6 discusses the results. Section 7 concludes the paper.

## 2. Materials and Methods

### 2.1. One-Bit DOA Signal Model

Consider a linear array with *M* omnidirectional antennas that receives *K* (K<M) independent narrowband far-field signals. The observed signal of the array at time *t* can be expressed as

(1) x(t)=A(θ)s(t)+n(t),t=1,⋯,L,

where

$$\begin{array}{cc} x(t) & ={\left[x_{1}\left(t\right),\cdots ,x_{M}\left(t\right)\right]}^{T}\in C^{M},\\ s(t) & ={\left[s_{1}\left(t\right),\cdots ,s_{K}\left(t\right)\right]}^{T}\in C^{K},\\ n(t) & ={\left[n_{1}\left(t\right),\cdots ,n_{M}\left(t\right)\right]}^{T}\in C^{M}, \end{array}$$

and s(t) and n(t) represent the signal vector and the noise vector, respectively. *L* is the number of snapshots. The noise n(t) is an independent identically distributed complex Gaussian circularly symmetric distribution with zero-mean and variance $\sigma_{n}^{2}$. The steering matrix is given by

$$\begin{array}{c} A\left(\theta \right)={\left[a\left(\theta_{1}\right),\ldots ,a\left(\theta_{K}\right)\right]}^{T}\in C^{M\times K}, \end{array}$$

which is composed of the steering vectors $a(\theta_{k})$ for k=1,2,…,K, and $\theta ={\left[\theta_{1},\theta_{2},\ldots ,\theta_{K}\right]}^{T}$ denotes DOA of *K* uncorrelated signals. In the ULA, the steering vector of the *k*-th signal can be represented as

$$\begin{array}{c} a\left(\theta_{k}\right)={\left[1,\exp\left(-j2\pi f_{k}\right)\cdots ,\exp\left(-j2\pi \left(M-1\right)f_{k}\right)\right]}^{T}\in C^{M}, \end{array}$$

where the spatial frequency $f_{k}$ is given by

(2) $$f_{k}=\frac{d\;\sin\theta_{k}}{\lambda },\;k=1,\cdots ,K,$$

*d* denotes the spacing between the adjacent antennas, and λ denotes the wavelength of the signal.

To introduce a one-bit quantized signal, the complex sign function is defined as

(3) $$\mathrm{signe}\left[x\right]=\frac{1}{\sqrt{2}}\left\{\mathrm{sign}[ℜ(x)]+j\;\mathrm{sign}[ℑ(x)]\;\right\},$$

where ℜ(x) and ℑ(x) denote the real and imaginary parts of *x*, respectively, and sign[·] represents the sign function for real numbers, which is defined as

$$\begin{array}{c} \mathrm{sign}\left[x\right]=\left\{\begin{array}{cc} 1 & x>0\\ -1 & x\leq 0 \end{array}\right.. \end{array}$$

After one-bit quantization, the received signal can be represented as

(4) y(t)=signe[x(t)].

For simplicity of notation, A(θ) is represented as A. Equations (1) and (4) can be rewritten for the multi-snapshot case as

(5) X=AS+N,

(6) Y=signe[X],

where

$$\begin{array}{cc} S & =\left[s\left(1\right),s\left(2\right),\ldots ,s\left(L\right)\right]={\left[s_{1},s_{2},\ldots ,s_{K}\right]}^{T}\in C^{K\times L},\\ N & =\left[n\left(1\right),n\left(2\right),\ldots ,n\left(L\right)\right]\in C^{M\times L},\\ X & =\left[x\left(1\right),x\left(2\right),\cdots ,x\left(L\right)\right]\in C^{M\times L},\\ Y & =\left[y\left(1\right),y\left(2\right),\cdots ,y\left(L\right)\right]\in C^{M\times L}. \end{array}$$

S and N represent the signal matrix and the noise matrix, respectively, X and Y represent the input and output of the one-bit ADC, respectively. This work aims to improve the resolution of the estimated DOAs from the one-bit observations Y.

### 2.2. Atomic Norm Minimization

The atomic norm minimization algorithm, as a sparse recovery algorithm, can achieve gridless DOA estimation. It leverages the principle of the Vandermonde decomposition of Toeplitz covariance matrices [26], and seeks the minimal combination of atomic sets within the continuous dictionary to effectively estimate the DOAs of a uniform linear array. Compared to grid-based DOA estimation algorithms, it can avoid the grid mismatch issue.

The atomic set of atomic norm minimization under the continuous frequency domain can be constructed as

(7) $$A=\{a\left(\theta \right)c\;|\;\theta \in \left[-90^{\circ },90^{\circ }\right],∥c{∥}_{2}=1,c\in C^{1\times L}\}.$$

Let the noise-free signal model of the array be expressed as

(8) $$B=AS=\sum_{k}^{K}a\left(\theta_{k}\right)s_{k}.$$

It is clear from (8) that B can be represented as the smallest number of atoms in the atomic set A, denoted by

(9) $$\begin{array}{cc} {\left||B|\right|}_{A,0} & =\inf\left\{K\;|\;B=\sum_{k}^{K}p_{k}a\left(\theta_{k}\right)c_{k},a\left(\theta_{k}\right)c_{k}\in A,p_{k}\geq 0\right\}\\ & =\inf\left\{K\;|\;B=\sum_{k}^{K}a\left(f\right)s_{k},s_{k}=p_{k}c_{k}\right\}. \end{array}$$

The ANM algorithm aims to solve the following optimization problem $\min_{B}{\left||B|\right|}_{A,0}$, which can be characterized as a rank minimization problem [27]

(10) $$\begin{array}{cc} \min_{u,B} & \mathrm{rank}\left(T(u)\right)\\ s.t. & \mathrm{tr}\left(B^{H}T{\left(u\right)}^{-1}B\right)<+\infty \\ \; & T(u)\geq 0, \end{array}$$

where T(·) denotes the Toeplitz transformation of the vector

(11) $$T\left(u\right)=\left[\begin{array}{cccc} u_{1} & u_{2} & \cdots & u_{M}\\ u_{2}^{H} & u_{1} & \cdots & u_{M-1}\\ \vdots & \vdots & \ddots & \vdots \\ u_{M}^{H} & u_{M-1}^{H} & \cdots & u_{1} \end{array}\right].$$

The conventional solution of the atomic-$\ell_{0}$ norm is relaxed into the conevx atomic-$\ell_{1}$ norm

(12) $$\begin{array}{cc} {\left||B|\right|}_{A} & =\inf\left\{\sum_{k}^{K}p_{k}\;|\;B=\sum_{k}^{K}p_{k}a\left(\theta_{k}\right)c_{k},a\left(\theta_{k}\right)c_{k}\in A,p_{k}\geq 0\right\}\\ & =\inf\left\{\sum_{k}^{K}p_{k}\;|\;B=\sum_{k}^{K}a\left(f\right)s_{k},s_{k}=p_{k}c_{k}\right\}. \end{array}$$

which indicates that model (12) can be reformulated as an SDP problem

(13) $$\begin{array}{cc} \min_{u,B} & \mathrm{tr}(T(u))\\ s.t. & \mathrm{tr}\left(B^{H}T{\left(u\right)}^{-1}B\right)<+\infty \\ & T(u)\geq 0. \end{array}$$

It can be observed that (13) approximates the rank norm in (10) using the trace norm, which is a loose approximation. However, the trace norm is suboptimal. It can be understood that the trace norm is the $\ell_{1}$-norm of the eigenvalues of T(u), and the $\ell_{1}$-norm is known to have a limited resolving capability [25]. Consequently, to achieve a high-resolution gridless one-bit DOA estimation, the next section will introduce a robust reweighted binary atomic norm minimization (robust RBANM) method by employing the log-det heuristic that better fits the rank function and considers the presence of noise.

## 3. Proposed Method

The BANM algorithm considers the sign consistency between Y and B, and uses linear constraints as the fidelity term. To achieve high resolution, we employ the atomic $\ell_{0}$ norm which has no limited resolution. Then, the optimization problem with one-bit atomic $\ell_{0}$ norm involving the linear constraints is given as

(14) $$\begin{array}{cc} \min_{u,B} & {\left||B|\right|}_{A,0}-\mu \mathrm{tr}\left(Y_{r}^{T}B_{r}\right)-\mu \mathrm{tr}\left(Y_{i}^{T}B_{i}\right)\\ s.t. & {∥X∥}_{F}=1, \end{array}$$

where μ is the regularization parameter, and ${∥B∥}_{F}=1$ is the normalization constraint which can reduce the optimization search space.

The linear constraints have certain robustness to noise; however, the $\ell_{0}$-norm is more sensitive to data than the $\ell_{1}$-norm, and using only the atomic $\ell_{0}$-norm and the linear constraints on Y and B may lead to inaccurate estimates. Therefore, to achieve a more robust high-resolution one-bit DOA estimation, the influence of noise should be considered. It is obvious that the one-bit ADC input X and output Y maintain sign consistency in the presence of noise

(15) $$Y_{r}⊙X_{r}\geq 0,$$

(16) $$Y_{i}⊙X_{i}\geq 0,$$

where $Y_{r}$ and $Y_{i}$ represent the real and imaginary parts of Y, respectively, and $X_{r}$ and $X_{i}$ represent the real and imaginary parts of X, respectively. The ⊙ symbol denotes the Hadamard product, and 0 represents a matrix with all elements equal to 0.

Based on the assumption of noise, the noise constraints can be reformulated as

(17) $$\mathrm{tr}\left\{{\left(X-AS\right)}^{H}{\left(\sigma^{2}I\right)}^{-1}\left(X-AS\right)\right\}<+\infty .$$

With the consistency between Y and the sign of X, the one-bit atomic $\ell_{0}$ norm optimization problem is formulated as

(18) $$\begin{array}{cc} \min_{u,B} & {\left||B|\right|}_{A,0}-\mu \mathrm{tr}\left(Y_{r}^{T}X_{r}\right)-\mu \mathrm{tr}\left(Y_{i}^{T}X_{i}\right)\\ s.t. & \mathrm{tr}\left\{{\left(X-B\right)}^{H}{\left(\sigma^{2}I\right)}^{-1}\left(X-B\right)\right\}<+\infty \\ \; & {∥X∥}_{F}=1. \end{array}$$

According to (10), the newly established one-bit atomic $\ell_{0}$ norm optimization problem (18) can be transformed into a rank minimization problem

(19) $$\begin{array}{cc} \min_{u,B} & \mathrm{rank}\left(T(u)\right)-\mu \mathrm{tr}\left(Y_{r}^{T}X_{r}\right)-\mu \mathrm{tr}\left(Y_{i}^{T}X_{i}\right)\\ s.t. & \mathrm{tr}\left(B^{H}T{\left(u\right)}^{-1}B\right)<+\infty \\ \; & \mathrm{tr}\left\{{\left(X-B\right)}^{H}{\left(\sigma^{2}I\right)}^{-1}\left(X-B\right)\right\}<+\infty \\ \; & T(u)\geq 0\\ \; & \sigma^{2}\geq 0\\ \; & {∥X∥}_{F}=1. \end{array}$$

As studied in [28,29], the log-det heuristic can better approximate the rank function

(20) $$\mathrm{rank}\left(T(u)\right)={∥\lambda ∥}_{0}\approx \sum_{m=1}^{M}\ln|\lambda_{m}+\epsilon |=\ln|T\left(u\right)+\epsilon I|,$$

where λ represents the eigenvalue vector of T(u), and $\lambda_{m}$ is the *m*-th largest eigenvalue of T(u). The parameter ϵ>0 can avoid $\ln|\lambda_{m}+\epsilon |$ and ln|T(u)+ϵI| being −∞. The smaller ϵ becomes, ln|T(u)+ϵI| approaches $\mathrm{rank}\left(T(u)\right)$.

Define two parameters $V\geq B^{H}T{\left(u\right)}^{-1}B$, $\Lambda \geq {\left(X-B\right)}^{H}{\left(\sigma^{2}I\right)}^{-1}\left(X-B\right)$. The first four constraints in (19) can be approximated by solving the optimization

(21) $$\begin{array}{cc} \min_{B} & \mathrm{tr}\left(V\right)+\mathrm{tr}\left(\Lambda \right)\\ s.t. & \left[\begin{array}{cc} T(u) & B\\ B^{H} & V \end{array}\right]\geq 0\\ \; & \left[\begin{array}{cc} \sigma^{2}I & X-B\\ {\left(X-B\right)}^{H} & \Lambda \end{array}\right]\geq 0. \end{array}$$

To reduce the number of variables, by replacing V+Λ with V and eliminating the variable B, we can also solve the following optimization

(22) $$\begin{array}{cc} \min_{B} & \mathrm{tr}\left(V\right)\\ s.t. & \left[\begin{array}{cc} T\left(u\right)+\sigma^{2}I & X\\ X^{H} & V \end{array}\right]\geq 0\\ \; & T(u)\geq 0\\ \; & \sigma^{2}\geq 0. \end{array}$$

Motivated by the log-det heuristic (20) and positive semidefinite transformation (22), (19) can be rewritten as

(23) $$\begin{array}{cc} \min_{u,B} & \ln|T\left(u\right)+\epsilon I|-\mu \mathrm{tr}\left(Y_{r}^{T}X_{r}\right)-\mu \mathrm{tr}\left(Y_{i}^{T}X_{i}\right)\\ s.t. & \left[\begin{array}{cc} T\left(u\right)+\sigma^{2}I & X\\ X^{H} & V \end{array}\right]\geq 0\\ \; & T(u)\geq 0\\ \; & \sigma^{2}\geq 0\\ \; & {∥X∥}_{F}=1. \end{array}$$

The optimization problem (23) is a non-convex problem. Since ln|T(u)+ϵI| is smooth over the positive semidefinite cone, local minimization methods can be used to minimize it. The common algorithm employed is the MM algorithm. The principle of the MM algorithm is to construct a majorant function, which is an upper bound of the objective function, and in each iteration step, the majorant function is minimized to obtain the next iteration point. A commonly used majorant function is the first-order Taylor expansion of the original objective function.

The term ln|T(u)+ϵI| can be approximated as

(24) $$\ln|T\left(u\right)+\epsilon I|=\ln|T\left(u^{\left(\ell \right)}\right)+\epsilon I|+\mathrm{tr}\left({\left(T(u^{\left(\ell \right)})+\epsilon I\right)}^{-1}T\left(u-u^{\left(\ell \right)}\right)\right).$$

Let $W={\left(T(u)+\epsilon I\right)}^{-1}$ and remove the constant term. The optimization problem at the (ℓ+1)th iteration can be rewritten as

(25) $$\begin{array}{cc} \min & \mathrm{tr}\left(W^{\left(\ell \right)}T\left(u\right)\right)-\mu \mathrm{tr}\left(Y_{r}^{T}X_{r}\right)-\mu \mathrm{tr}\left(Y_{i}^{T}X_{i}\right)\\ s.t. & \left[\begin{array}{cc} T\left(u\right)+\sigma^{2}I & X\\ X^{H} & V \end{array}\right]\geq 0\\ \; & T(u)\geq 0\\ \; & \sigma^{2}\geq 0\\ \; & {∥X∥}_{F}=1. \end{array}$$

The robust RBANM DOA estimation optimization problem is established. The approach to solve this optimization problem is introduced in the following section.

## 4. Alternating Direction Method of Multipliers (ADMM) for the Proposed Method

In this section, the ADMM algorithm is employed to solve the optimization problem (25). ADMM is a computational method for solving convex optimization problems with separable structures. It has a fast processing speed and good convergence performance. The fundamental principle underlying the ADMM algorithm is to optimize the original variables in an alternating manner by constructing an augmented Lagrangian function. The augmented Lagrangian function of (25) can be written as

(26) $$\begin{array}{cc} L(u,X,\sigma^{2},V,Z,Q)= & \mathrm{tr}\left(W^{\left(\ell \right)}T\left(u\right)\right)+\mathrm{tr}\left(V\right)-\mu \mathrm{tr}\left(Y_{r}^{T}X_{r}\right)-\mu \mathrm{tr}\left(Y_{i}^{T}X_{i}\right)\\ \; & +\mathrm{tr}\left\{\left(Z-\left[\begin{array}{cc} T\left(u\right)+\sigma^{2}I & X\\ X^{H} & V \end{array}\right]\right)Q\right\}+\frac{\rho }{2}{∥Z-\left[\begin{array}{cc} T\left(u\right)+\sigma^{2}I & X\\ X^{H} & V \end{array}\right]∥}_{F}^{2}, \end{array}$$

where Q is the Lagrangian multiplier and ρ>0 is a penalty parameter, and an auxiliary variable Z is newly introduced

(27) $$Z=\left[\begin{array}{cc} T\left(u\right)+\sigma^{2}I & X\\ X^{H} & V \end{array}\right]\geq 0.$$

Then, the updating iterations are as follows

(28) $$\begin{array}{cc} u^{\left[l+1\right]},X^{\left[l+1\right]},V^{\left[l+1\right]}= & \arg\min L(u,X,\sigma^{2[l]},V,Z^{\left[l\right]},Q^{\left[l\right]}), \end{array}$$

(29) $$\begin{array}{cc} \sigma^{2[l+1]}= & \arg\min L(u^{\left[l+1\right]},X^{\left[l+1\right]},\sigma^{2},V^{\left[l+1\right]},Z^{\left[l\right]},Q^{\left[l\right]}), \end{array}$$

(30) $$\begin{array}{cc} Z^{\left[l+1\right]}= & \arg\min L(u^{\left[l+1\right]},X^{\left[l+1\right]},\sigma^{2[l+1]},V^{\left[l+1\right]},Z,Q^{\left[l\right]}), \end{array}$$

(31) $$\begin{array}{cc} Q^{\left[l+1\right]}= & Q^{\left[l\right]}+\left(Z^{\left[l+1\right]}-\left[\begin{array}{cc} T\left(u^{\left[l+1\right]}\right)+\sigma^{2[l+1]}I & X^{\left[l+1\right]}\\ X^{H[l+1]} & V^{\left[l+1\right]} \end{array}\right]\right), \end{array}$$

where [l] is set as the *l*th iteration of the inner loop and (*ℓ*) is set as the *ℓ*th iteration of the outer loop to discriminate the iteration of inner loop and outer loop.

Let Z and Q be partitioned as

(32) $$Z=\left[\begin{array}{cc} Z_{0} & Z_{2}\\ Z_{2}^{H} & Z_{1} \end{array}\right]=\left[\begin{array}{cc} Z_{0} & Z_{r}+jZ_{i}\\ Z_{r}^{H}+jZ_{i}^{H} & Z_{1} \end{array}\right],$$

(33) $$Q=\left[\begin{array}{cc} Q_{0} & Q_{2}\\ Q_{2}^{H} & Q_{1} \end{array}\right]=\left[\begin{array}{cc} Q_{0} & Q_{r}+jQ_{i}\\ Q_{r}^{H}+jQ_{i}^{H} & Q_{1} \end{array}\right],$$

where $Z_{0}$, $Q_{0}\in C^{M\times M}$, $Z_{1}$, $Q_{1}\in C^{L\times L}$, and $Z_{2}$, $Z_{r}$, $Z_{i}$, $Q_{2}$, $Q_{r}$,$Q_{i}\in C^{M\times L}$. $Z_{r}$ and $Z_{i}$ represent the real part and imaginary part of $Z_{2}$, and $Q_{r}$ and $Q_{i}$ represent the real part and imaginary part of $Q_{2}$. Taking the derivative of (26) with respect to each parameter yields

{(34)∂L∂u=T∗W1(ℓ)−Q0−ρ[Z0−T(u)−σ2I],(35)∂L∂σ2=−D(Q0)−ρ[D(Z0)−D(T(u))−Mσ2],(36)∂L∂Xr=−μYr−2Qr−2ρZr−Xr,(37)∂L∂Xi=−μYi−2Qi−2ρZi−Xi,(38)∂L∂V=I−Q1−ρZ1−V,(39)∂L∂Z=Q+ρZ−T(u)+σ2IXXHV,

where $T^{*}\left(\cdot \right)$ is the Toeplitz adjoint operator, D(·) is the operator that calculates the sum of the diagonal elements of matrix.

Let (34) and (35) be equal to zero. Then, we have

(40) $$u=T^{*}\left(Z_{0}\right)-\sigma^{2}e_{1}+\frac{1}{\rho }T^{*}\left(Q_{0}\right)+\frac{1}{\rho }T^{*}\left(W\right),$$

(41) $$\sigma^{2}=\frac{1}{M}D\left(Z_{0}\right)-u_{1}+\frac{1}{\rho }\frac{1}{M}D\left(Q_{0}\right),$$

where $e_{1}$ represents the unit vector with the first element equal to 1 and the remaining elements to 0. $u_{1}$ is the first element of u. Substitute (41) into (40), and let (36)–(38) be equal to zero. The variables in (28) are given as

{(42)u[l+1]=T∗Z0[l]−D(Z0[l])+1ρ(Q0[l]−D(Q0[l]))−1ρW1(ℓ)+u1[l]I+,(43)Xr[l+1]=Zr[l]+1ρQr[l]+μρYr,(44)Xi[l+1]=Zi[l]+1ρQi[l]+μρYi,(45)V[l+1]=Z1[l]+1ρQ1[l]−1ρI.

where ${\left\{\cdot \right\}}_{+}$ represents the operator of the positive semidefinite cone. The operator decomposes the matrix eigenvalues and sets all negative eigenvalues to zero, which forces the matrix onto the positive definite cone. The variable $\sigma^{2}$ in (41) can be updated with the closed form as below

(46) $$\sigma^{2[l+1]}={\left[\frac{1}{M}D\left(Z_{0}^{\left[l\right]}\right)-u_{1}^{\left[l+1\right]}+\frac{1}{\rho }\frac{1}{M}D\left(Q_{0}^{\left[l\right]}\right)\right]}_{+},$$

where ${\left[\cdot \right]}_{+}$ is an operator that sets negative values to 0 and leaves non-negative values unchanged. Let (39) be equal to zero; with the constraint that Z is a positive semidefinite matrix, it can be calculated by

(47) $$Z^{\left[l+1\right]}={\left\{\left[\begin{array}{cc} T\left(u^{\left[l+1\right]}\right)+\sigma^{2[l+1]}I & X^{\left[l+1\right]}\\ X^{H[l+1]} & V^{\left[l+1\right]} \end{array}\right]-\frac{1}{\rho }Q^{\left[l\right]}\right\}}_{+},$$

The iterative formula for Q can be written as

(48) $$Q^{\left[l+1\right]}=Q^{\left[l\right]}+\rho \left(Z^{\left[l+1\right]}-\left[\begin{array}{cc} T\left(u^{\left[l+1\right]}\right)+\sigma^{2[l+1]}I & X^{\left[l+1\right]}\\ X^{H[l+1]} & V^{\left[l+1\right]} \end{array}\right]\right).$$

By iterative optimization, existing algorithms such as MUSIC, root-MUSIC, and rotation-invariance methods can utilize the pseudo-covariance $T(\hat{u})$ to estimate DOAs.

## 5. Results

In this section, the proposed robust RBANM is compared with existing methods, such as one-bit MUSIC [30] and BANM [13,14]. Additionally, to demonstrate the robustness of the proposed algorithm, the noiseless one-bit atomic $\ell_{0}$ norm, i.e., the optimization problem (14), is also compared with the proposed method, and it is referred to as RBANM here. To ensure consistency, all four algorithms utilize the MUSIC algorithm for DOA estimation. The estimates T(u) obtained from BANM, RBANM, and robust RBANM are used to perform eigendecomposition, extract the noise subspace, and apply the MUSIC algorithm to obtain the spectrum, while the one-bit MUSIC algorithm utilizes the quantized signal covariance $R_{y}=YY^{H}/L$ to obtain the spectrum. For the BANM, RBANM, and robust RBANM algorithms, the iterative process is terminated when the Frobenius norm of the difference between two consecutive estimates T(u) is less than $10^{-2}$. The initial value of ϵ is set to 1, and then a strategy is adopted that, in each iteration, $\epsilon^{\left(\ell \right)}=\frac{\epsilon^{\left(\ell -1\right)}}{2}$ until $\epsilon^{\left(\ell \right)}\leq 10^{-2}$ is reached. The regularization parameter μ is set to 1. The signals $s_{k}$, k=1,2,⋯,K are set to have equal energy. The signal-to-noise ratio (SNR) is defined as

(49) $$\mathrm{SNR}=10\log_{10}\left(\frac{\sum_{k=1}^{K}\sigma_{k}^{2}}{\sigma_{n}^{2}}\right).$$

**Figure 1:**
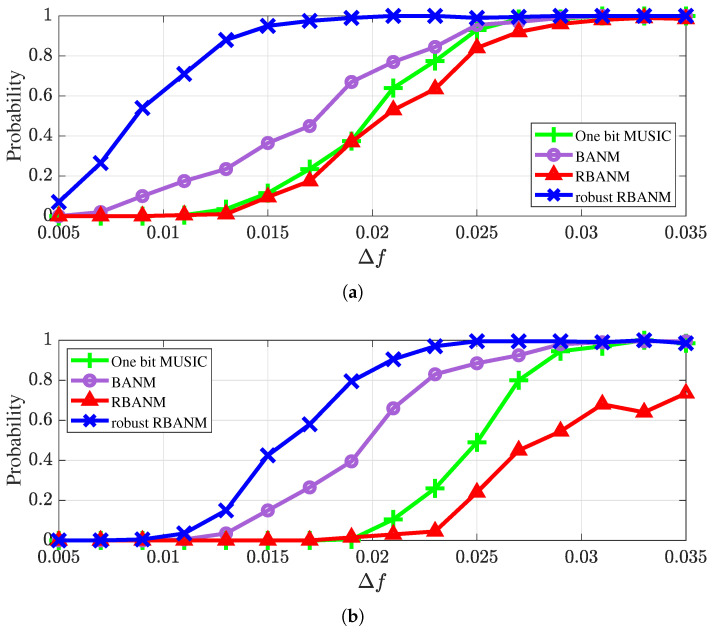
The identification probabilities with different spatial frequency separation Δf under M=40, L=5, and SNR =10 dB. (**a**) In the case of two signals at 0.1 and 0.1 + Δf. (**b**) In the case of three signals at 0.1, 0.1 + Δf, and 0.1 + 2Δf.

First, the identification probabilities of the four algorithms with different spatial frequency separation Δf under a different number of signals with L=5 snapshots are shown in Figure 1. The signal identification criterion is that the DOA estimates from the four methods are within f±Δf/2. If all signals are correctly estimated, the trial is counted as 1, otherwise 0. The results are averaged over 500 Monte Carlo trials. The simulation environment is a ULA with M=40 sensors, and SNR is set to 10 dB. Figure 1a illustrates the identification probabilities in the case of two signals, where the spatial frequencies of the signals are 0.1 and 0.1+Δf, respectively. It can be seen that the proposed robust RBANM algorithm consistently has the highest identification probability, achieving a probability of 1 when Δf is greater than 0.021, while MUSIC and BANM reach an identification probability of 1 when Δf is greater than 0.029. Figure 1b illustrates the identification probabilities in the case of three signals, where the spatial frequencies of the signals are 0.1, 0.1+Δf, and 0.1+2Δf, respectively. It can be seen that the robust RBANM algorithm achieves an identification probability of 1 when Δf is greater than 0.025, while MUSIC and BANM reach an identification probability of 1 when Δf is greater than 0.033.

**Figure 2:**
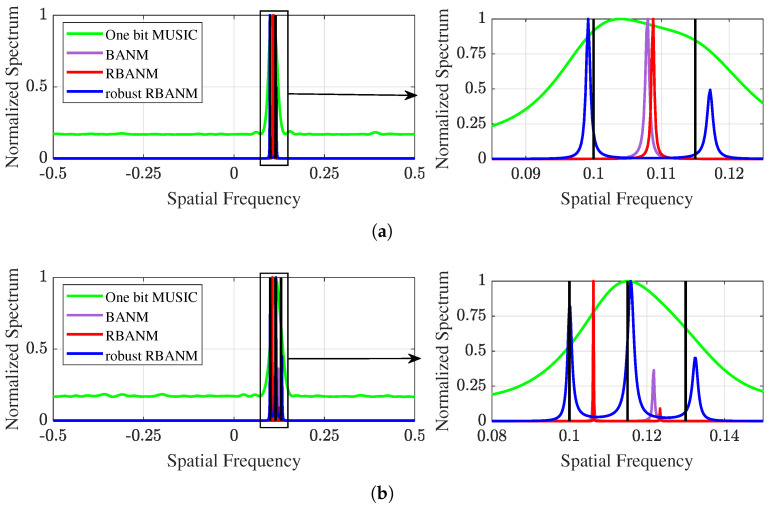
Two illustrative examples with M=40, L=5, and SNR =10 dB. (**a**) The signals are at 0.1 and 0.115. The left spectrum is shown on the interval [−0.5,0.5], while the right is shown on [0.085,0.125]. (**b**) The signals are at 0.1, 0.115, and 0.13. The left spectrum is shown on the interval [−0.5,0.5], while the right is shown on [0.08,0.15].

**Figure 3:**
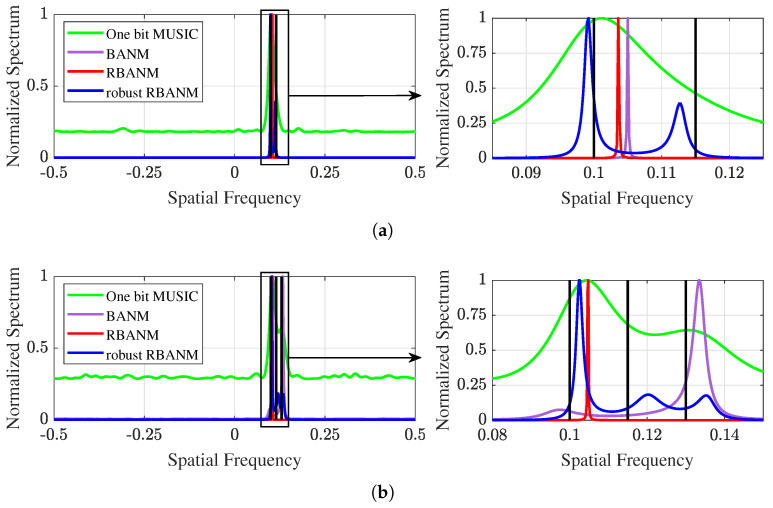
Two illustrative examples with M=40, L=5, and SNR =5 dB. (**a**) The signals are at 0.1 and 0.115. The left spectrum is shown on the interval [−0.5,0.5], while the right is shown on [0.085,0.125]. (**b**) The signals are at 0.1, 0.115, and 0.13. The left spectrum is shown on the interval [−0.5,0.5], while the right is shown on [0.08,0.15].

Then, Figure 2 presents two illustrative examples corresponding to Figure 1a and Figure 1b, respectively. The black vertical lines indicate the true signal directions. Figure 2a shows the spectrum obtained by the four algorithms when the two signals are located at 0.1 and 0.115. The proposed robust RBANM successfully identifies the two signals at Δf=0.015, while the other algorithms cannot. Figure 2b provides an example where the proposed algorithm successfully identifies the three signals with spatial frequencies at 0.1, 0.115, and 0.13, while the other algorithms fail to completely distinguish the three signals. It can be observed that the robust RBANM algorithm can clearly generate three distinct peaks, while BANM only generates two peaks, and the other two algorithms generate only one peak. Similar examples are shown in Figure 3. All simulation conditions are the same, except for SNR = 5 dB. It can be observed from Figure 3 that even at low SNR, the robust RBANM algorithm is still able to estimate the signals.

**Figure 4:**
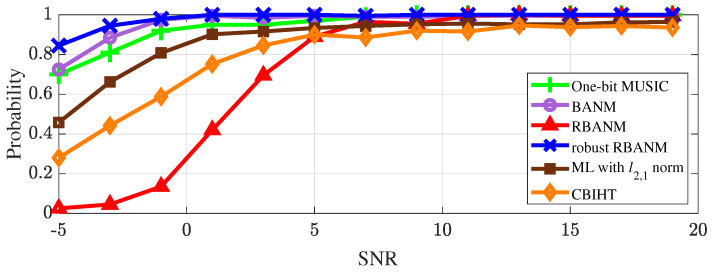
The identification probability of the four algorithms under different SNRs with M=40 and L=5. The signals are at f=0.1,0.13.

**Figure 5:**
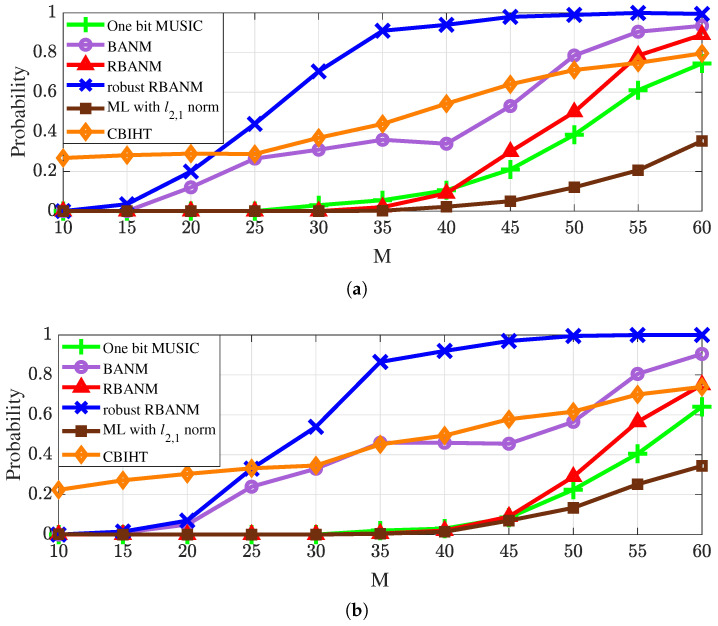
The identification probabilities under a different number of sensors *M* with L=5. The signals are at f=0.1,0.115. (**a**) SNR =10 dB. (**b**) SNR =5 dB.

Finally, the performance of the proposed algorithm under different signal-to-noise ratios and array element numbers is shown in Figure 4 and Figure 5. Additionally, algorithms that combine maximum likelihood with the $\ell_{2,1}$ norm [17] and the CBIHT algorithm [11] are added for performance comparison.

Figure 4 presents the robustness of the four algorithms under different SNRs, and shows the identification probabilities of the four algorithms when the two signals are located at 0.1 and 0.13. The results are averaged over 500 Monte Carlo trials. It can be seen that all algorithms, except for RBANM, achieve the identification probabilities of 0.7 for the two signals when SNR is above 1 dB. In contrast, RBANM is affected by noise and can hardly accurately detect the signals at −5 dB SNR, and it can only completely identify the two signals when SNR reaches 11 dB.

Figure 5 presents the identification probabilities under a different number of sensors *M*. The simulation condition set as snapshots L=5 and the signals are at f=0.1,0.115. The SNRs of Figure 5a and Figure 5b are set as 10 dB and 5 dB, respectively. The results are averaged over 500 Monte Carlo trials. As shown in Figure 5, all algorithms can achieve higher detection probabilities by increasing the number of sensors, while the proposed robust RBANM consistently maintains the highest identification probability when the number of sensors *M* is more than 25.

## 6. Discussion

This paper proposes a high-resolution and robust binary atomic norm minimization algorithm. The proposed algorithm is compared to several recent one-bit DOA studies in Section 5, considering the detection performance under different signal spatial frequency separations, varying signal-to-noise ratios, and different numbers of sensors.

It can be observed that the proposed algorithm achieves high-resolution and robust estimation, significantly improving the resolution capability compared to BANM. Furthermore, as shown in Figure 3b and Figure 5a, it maintains good detection performance even at lower SNRs. For instance, in the noisy environment with an SNR of 5 dB, with M=50 sensors, the algorithm accurately identifies signals with a spatial frequency difference of 0.015, while the other algorithms fail to identify them.

However, the proposed algorithm involves nested iterations, resulting in higher computational complexity. In future work, we will seek alternatives to the ADMM algorithm or solutions that avoid nested iterations.

## 7. Conclusions

In this paper, a novel approach for high-resolution and robust one-bit DOA estimation is proposed. This method has two significant advantages. First, the proposed method achieves high-resolution DOA estimation by using the log-det heuristic instead of the conventional atomic $\ell_{1}$ norm minimization, which may lead to inaccurate estimates, to approximate the binary atomic $\ell_{0}$ norm minimization problem, compared to the existing BANM algorithm. Second, by adding a noise constraint, the proposed method improves robustness against noise sensitivity inherent in atomic $\ell_{0}$ norm methods, resulting in more reliable DOA estimation. Simulation results demonstrate the effectiveness of the proposed method.

## Data Availability

The original contributions presented in the study are included in the article, further inquiries can be directed to the corresponding author.

## Abbreviations

The following abbreviations are used in this manuscript:

ADC	Analog-to-Digital Converter
ADMM	Alternating Direction Method of Multipliers
ANM	Atomic Norm Minimization
BANM	Binary Atomic Norm Minimization
DOA	Direction-Of-Arrival
MM	Majorization Minimization
MUSIC	MUltiple SIgnal Classification
RBANM	Reweighted Binary Atomic Norm Minimization
SDP	SemiDefinite Programming
SNR	Signal-to-Noise Ratio
ULA	Uniform Linear Array

## References

[1] Dey N., Ashour A.S. (2018). Direction of Arrival Estimation and Localization of Multi-Speech Sources.

[2] Ho T., McWhirter J., Nehorai A., Nickel U., Ottersten B., Steinberg B., Stoica P., Viberg M., Zhu Z. (2013). Radar Array Processing.

[3] Walden R.H. (1999). Analog-to-digital converter survey and analysis. IEEE J. Sel. Areas Commun..

[4] Lu L., Li G.Y., Swindlehurst A.L., Ashikhmin A., Zhang R. (2014). An overview of massive MIMO: Benefits and challenges. IEEE J. Sel. Top. Signal Process..

[5] Ruan N., Wang H., Wen F., Shi J. (2022). DOA estimation in B5G/6G: Trends and challenges. Sensors.

[6] Liu C.L., Vaidyanathan P. One-bit sparse array DOA estimation. Proceedings of the 2017 IEEE International Conference on Acoustics, Speech and Signal Processing (ICASSP).

[7] Singh J., Dabeer O., Madhow U. (2009). On the limits of communication with low-precision analog-to-digital conversion at the receiver. IEEE Trans. Commun..

[8] Mezghani A., Nossek J.A. Analysis of Rayleigh-fading channels with 1-bit quantized output. Proceedings of the 2008 IEEE International Symposium on Information Theory.

[9] Stoica P., Shang X., Cheng Y. (2021). The Cramér–Rao bound for signal parameter estimation from quantized data [Lecture Notes]. IEEE Signal Process. Mag..

[10] Bar-Shalom O., Weiss A.J. (2002). DOA estimation using one-bit quantized measurements. IEEE Trans. Aerosp. Electron. Syst..

[11] Stöckle C., Munir J., Mezghani A., Nossek J.A. 1-bit direction of arrival estimation based on compressed sensing. Proceedings of the 2015 IEEE 16th International Workshop on Signal Processing Advances in Wireless Communications (SPAWC).

[12] Huang X., Xiao P., Liao B. One-bit direction of arrival estimation with an improved fixed-point continuation algorithm. Proceedings of the 2018 10th International Conference on Wireless Communications and Signal Processing (WCSP).

[13] Zhou C., Zhang Z., Liu F., Li B. (2017). Gridless compressive sensing method for line spectral estimation from 1-bit measurements. Digit. Signal Process..

[14] Wei Z., Wang W., Dong F., Liu Q. (2020). Gridless one-bit direction-of-arrival estimation via atomic norm denoising. IEEE Commun. Lett..

[15] Tang W.G., Jiang H., Zhang Q. (2022). One-bit gridless DOA estimation with multiple measurements exploiting accelerated proximal gradient algorithm. Circuits Syst. Signal Process..

[16] Feng L., Huang L., Li Q., He Z.Q., Chen M. (2023). An off-grid iterative reweighted approach to one-bit direction of arrival estimation. IEEE Trans. Veh. Technol..

[17] Chen M., Li Q., Li X.P., Huang L., Rihan M. (2024). One-Bit DoA Estimation for Deterministic Signals Based on *ℓ*_2,1_-Norm Minimization. IEEE Trans. Aerosp. Electron. Syst..

[18] Marques E.C., Maciel N., Naviner L., Cai H., Yang J. (2018). A review of sparse recovery algorithms. IEEE Access.

[19] Malioutov D., Cetin M., Willsky A.S. (2005). A sparse signal reconstruction perspective for source localization with sensor arrays. IEEE Trans. Signal Process..

[20] Bhaskar B.N., Tang G., Recht B. (2013). Atomic norm denoising with applications to line spectral estimation. IEEE Trans. Signal Process..

[21] Yang Z., Xie L. (2015). Enhancing sparsity and resolution via reweighted atomic norm minimization. IEEE Trans. Signal Process..

[22] Wu X., Zhu W.P., Yan J. (2018). A high-resolution DOA estimation method with a family of nonconvex penalties. IEEE Trans. Veh. Technol..

[23] Sheng S., Chen P., Yao Y., Wu L., Chen Z. (2021). Atomic network-based DOA estimation using low-bit ADC. Electronics.

[24] Jia T., Liu H., Gao C., Yan J. (2024). Bayesian Direction of Arrival Estimation using Atomic Norm Minimization with Prior Knowledge. IEEE Trans. Aerosp. Electron. Syst..

[25] Fernandez-Granda C. (2016). Super-resolution of point sources via convex programming. Inf. Inference J. IMA.

[26] Yang Z., Xie L. (2016). Exact joint sparse frequency recovery via optimization methods. IEEE Trans. Signal Process..

[27] Tang G., Bhaskar B.N., Shah P., Recht B. (2013). Compressed sensing off the grid. IEEE Trans. Inf. Theory.

[28] Fazel M., Hindi H., Boyd S.P. Log-det heuristic for matrix rank minimization with applications to Hankel and Euclidean distance matrices. Proceedings of the 2003 American Control Conference.

[29] Shen L., Suter B.W. (2016). One-bit compressive sampling via *ℓ*_0_ minimization. EURASIP J. Adv. Signal Process..

[30] Huang X., Liao B. (2019). One-bit MUSIC. IEEE Signal Process. Lett..

